# The rotavirus vaccine development pipeline

**DOI:** 10.1016/j.vaccine.2017.03.076

**Published:** 2019-11-28

**Authors:** Carl D. Kirkwood, Lyou-Fu Ma, Megan E. Carey, A. Duncan Steele

**Affiliations:** Enteric & Diarrheal Diseases, Global Health, Bill & Melinda Gates Foundation, Seattle, WA, USA

**Keywords:** Rotavirus, Vaccines, Non-replicating

## Abstract

Rotavirus disease is a leading global cause of mortality and morbidity in children under 5 years of age. The effectiveness of the two globally used oral rotavirus vaccines quickly became apparent when introduced into both developed and developing countries, with significant reductions in rotavirus-associated mortality and hospitalizations. However, the effectiveness and impact of the vaccines is reduced in developing country settings, where the burden and mortality is highest.

New rotavirus vaccines, including live oral rotavirus candidates and non-replicating approaches continue to be developed, with the major aim to improve the global supply of rotavirus vaccines and for local implementation, and to improve vaccine effectiveness in developing settings. This review provides an overview of the new rotavirus vaccines in development by developing country manufacturers and provides a rationale why newer candidates continue to be explored. It describes the new live oral rotavirus vaccine candidates as well as the non-replicating rotavirus vaccines that are furthest along in development.

## Graphical abstract

**Figure:**
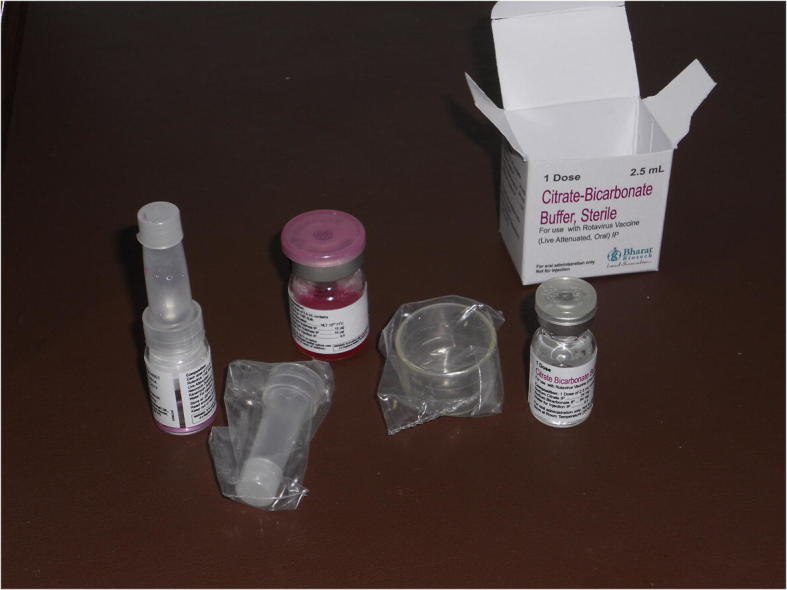

## 1 Introduction

While diarrheal diseases remain one of the leading infectious disease causes of Under-5 child mortality and morbidity [1], rotavirus infection is well characterized as the major pathogen associated with moderate-to-severe diarrhea in young children [2], [3]. It is a “democratic” infection and all children are susceptible to the disease regardless of socio-economic status; however, the greatest disease burden occurs in developing settings, predominantly in sub-Saharan Africa and south-east Asia [4]. The most recent mortality estimates based on Under-5 mortality data from 2013 and global rotavirus surveillance data from 2014, suggests that there are at least 215,000 (range 197,000–233,000) rotavirus deaths in children under 5 years of age globally, with 56% of deaths in sub-Saharan Africa and 22% in India alone [5].

Rotaviruses are excreted in enormous numbers in the stool, with more than 10^11^ particles per gram being described [6], and are transmitted through the fecal-oral route. Rotavirus is very stable in feces and can remain viable for days at room temperature, making them extremely resilient and readily transmitted in many settings, including maternity units, hospital wards and day care centers for young children [6]. There have also been reports of likely aerosol spread of rotavirus particle, possibly linked to the projectile vomiting early in infection and the explosive nature of the acute watery diarrhea. However, it is clear that rotaviruses replicate in the enterocytes of the small intestine and thus the virus must transit to the small intestine to cause the symptomatic disease. After a short incubation period of 24–48 h, projectile vomiting precedes watery diarrhea in more than half the cases [6], which is then followed by 5–7 days of acute watery diarrhea which can result in rapid dehydration; fever occurs in about a third of children [7]. Natural history studies have indicated that natural immunity gained by wildtype infection offers both some degree of protection from future symptomatic infections and that repeated exposures broaden a heterotypic immune response to different viral strains [8]. This has been the essential premise on which live, attenuated oral rotavirus vaccines have been developed over the past 30 years.

## 2 Rotavirus disease is a vaccine preventable disease

Two live oral rotavirus vaccines have been WHO prequalified and are commercially available globally. The current vaccines, RotaTeq® (a pentavalent human-bovine reassortant vaccine, Merck & Co., West Point, PA, USA) and Rotarix™ (a human monovalent G1P[8] vaccine, GlaxoSmithKline Biologicals, Rixensart, Belgium) are licensed in more than 100 countries. In 2009, the WHO Strategic Advisory Group of Experts (SAGE) recommended the inclusion of rotavirus vaccines into the national immunization program of all countries, in particularly those where diarrheal disease is a major health problem [9]. To date, these two rotavirus vaccines have been introduced into the national immunization program of 82 countries, including both high-income countries (HIC), upper-middle-income (UMIC) and low-middle income countries (LMIC). Currently, 40 Gavi-eligible countries have introduced the vaccines into their national immunization programs with support from the Gavi Alliance and UNICEF - including 27 countries from sub-Saharan Africa, 5 from the Americas, 5 from Europe and 3 from East Mediterranean/Middle East region. Furthermore, regional vaccine programs have been initiated in India, The Philippines, Thailand and Canada. In addition, several countries (Nigeria, Pakistan, Central African Republic and Cote d’Ivoire) have obtained Gavi approval for funding to support the introduction of rotavirus vaccine [10], [11].

In clinical trials, both Rotarix™ and RotaTeq® have proven to be highly efficacious in HIC and UMIC at reducing severe rotavirus disease (>85%) [12], [13], [14], [15], [16]. Efficacy studies conducted in LMIC and LIC in Africa and Asia revealed that both vaccines were less efficacious against both severe rotavirus disease and any rotavirus gastroenteritis [17], [18], [19]. Nevertheless, it was these studies that led to a global recommendation by WHO SAGE for the use of the vaccines based on the clear public health impact of the vaccines [9].

In all settings, developed or developing, where rotavirus vaccines have been introduced a real public health impact has consistently been observed, with significant reductions in rotavirus related mortality, severe rotavirus disease and all cause diarrheal hospitalizations in children under the age of 5 years [20], [21]. However, the clinical efficacy and subsequent vaccine effectiveness and public health impact is consistently lower in developing country settings [20], [21], [22].

Vaccine effectiveness and impact studies conducted in high income settings, such as USA, Australia and Europe have consistently reported rates of 79–100% depending on the location and vaccine coverage [23], [24]. Countries within Latin America were some of the first adopters of rotavirus vaccines. A recent review and meta-analysis by Santos et al. [25] illustrated that in LIC and LMIC settings in Latin America, the early and widespread use of rotavirus vaccine has resulted in significant reduction of rotavirus-associated deaths and rotavirus hospitalizations. The meta-analysis of the numerous studies on the impact of Rotarix™ and RotaTeq® vaccines estimated that an overall vaccine effectiveness of 53% against rotavirus infections, 73% against rotavirus related hospitalizations and 74% against severe rotavirus disease was observed [25].

However, the bulk of rotavirus disease, including hospitalizations and deaths occur in low income settings in Africa and Asia. As indicated earlier, the limited clinical vaccine efficacy studies showed a substantially lower efficacy in these high disease burden low income settings. Nevertheless, and importantly, studies of vaccine effectiveness conducted in LIC or LMIC countries have all shown a real impact by reduction in rotavirus associated- and all cause diarrhea hospitalizations.

In Latin America, countries including Nicaragua and Bolivia, and in Africa; Malawi, Ghana, Botswana, Rwanda, and Zambia have successfully demonstrated reductions in rotavirus associated hospitalizations (45–75%) (Table 1) [26], [27], [28], [29], [30], [31], [32], [33]. In a number of studies in these countries however, a decline in vaccine effectiveness in the second year of life has been reported. The post licensure effectiveness studies conducted in countries such as Malawi, Moldova and Nicaragua have shown a significant reduction in effectiveness in the second year of life (Table 1) [31]. Additional evidence is required to confirm the extent of vaccine waning, in both African and Asian low income and LMIC settings. The vaccine effectiveness and impact studies from low income settings offers genuine hope that severe rotavirus disease can be significantly reduced, however, there are concerns that the protection is not as enduring or complete through the second year of life (see Table 2).

**Table 1:** Rotavirus vaccine effectiveness studies.

Country	Vaccine/year introduced	VE against RV hospitalization (full vaccination course, Vesikari > 11)	Change in VE between age <12 mo to >12 mo
Nicaragua	2006 - RotaTeq	Overall VE all – 45% (25–59%) (G2P4-63%, G1P8-42%; G3P8-23%)	Yes (statistically significant)
		6–11 mo – G2- 68%; G1- 71%; G3 - 40%	
		>12 mo - G2- 60%; G1- 25%; G3 - 22%	
		VE decline with age/genotype	
Rwanda	2012 - RotaTeq	Overall VE all – 75% (31–91%) (6–36 mo)	No
		6–11 mo – 65% (−80 to 93%)	
		>12 mo – 81% (25–95%)	
Bolivia	2008 – Rotarix	Overall VE all – 59% (37–73%) (2–59 mo)	
		2–11 mo – 76% (50–89%)	
		>12 mo – 45% (0–70%)	
Malawi	2012 – Rotarix	Overall VE all- 58.3% (20–78%) (0–59 mo)	Yes (statistically significant)
		(G1P8 – 82.1 (44–94); G2P4 – 34.9% (−135 to 82%)	
		<12 mo – 70.6% (33–87%)	
		12–23 mo – 31.7% (−140 to 80%)	
		24–31 mo – 28.8% (−147 to 79%)	
Moldova	2012 – Rotarix	Overall VE all – 79% (62–88%) (6–59 mo)	Yes (not statistically different)
		6–11 mo – 84% (67–92%)	
		12–23 mo – 46% (−16 to 75%)	
Armenia	2012 – Rotarix	Overall VE all – 62% (36–77%) (6–23 mo)	No
		6–11 mo - 68% (24–86%)	
		12–23 mo – 60% (20–80%)	
Botswana	2012 – Rotarix	Overall VE all - 54% (23–73%) (4–59 mo)	No
		4–11 mo – 52% (8–75%)	
		>12 mo – 67% (8–89%)

**Table 2:** Live oral rotavirus vaccines under clinical development.

Candidate	Producer	Strain	Characteristics	Route	Recent findings
RV3-BB	PT Bio Farma, Indonesia (MCRI, Australia)	Human neonatal G3P[6]	Frozen presentation, ongoing evaluation of a liquid product	Oral	Phase 2a immunogenicity: 76% (New Zealand)
					Phase 2b immunogenicity & efficacy underway in Indonesia
Bovine-human reassortants	Serum Institute of India, Pune	Pentavalent combination (G1-4, G9)	First presentation – lyo, liquid presentation under evaluation	Oral	Phase 3 efficacy of lyophilized product completed, awaiting results
G1P[5] G2P[5] G3P[5] G4P[5] G8P[5] G9P[5] and G6P[5] parent strain	Shantha Biotechnic, Hyderabad	Tetravalent combination (G1-4)	Liquid presentation	Oral	Phase 3 non-inferiority study completed
	Wuhan Institute of Biological Products, China	Hexavalent combination (G1-4, G8, G9)	Liquid presentation	Oral	Phase 1: age descending study approved in China, adult safety completed
	Institute Butantan, Brazil	Pentavalent combination (G1-4, G9)	Liquid presentation	Oral	Phase 1 safety in adults completed

It is unclear why vaccine take and subsequent vaccine effectiveness is lower in LIC and LMIC settings, however, a range of factors have been suggested. The higher transmission rates of rotavirus infection in developing settings, variations in gut microbiota, and host mucosal factors, such as the presence of breast milk constituents at the time of immunization, as well as other host factors (eg. HBGA and Lewis secretor antigens) may interfere with one or more stages of rotavirus vaccine take and resultant immune responses [34], [35], [36]. Studies are currently underway in multiple sites to help elucidate what the major mechanisms of reduced effectiveness are and if there are programmatic solutions.

## 3 Progress for new live-attenuated, oral rotavirus licensed vaccines

Besides the two commercial and WHO-prequalified vaccines described above, three additional live, attenuated orally administered rotavirus vaccines have obtained national licensure in the country of manufacture, using the same rationale and development strategies as the two commercial vaccines by the multi-national companies. The new oral rotavirus vaccines will provide additional options to the global community, once prequalified by WHO, as well as provide indigenous vaccines for several countries who desire their own rotavirus vaccine (eg India, Vietnam and Indonesia). The new oral rotavirus vaccines should aim to achieve similar vaccine efficacy and effectiveness as the two globally utilized rotavirus vaccines. Development of these additional oral candidates will help to ensure an adequate global vaccine supply and vaccine diversity. The diversity of manufacturers and market pressure, should assist to keep the cost of vaccine low for those countries with the highest burden of disease. Each of these new oral rotavirus vaccine need to ensure they develop products with an acceptable presentation for use in Gavi-eligible countries and LMICs, with a particular focus on programmatic issues such as reducing the package footprint within the cold chain, carry a vaccine vial monitor (VVM), and providing a preferred product presentation (eg. liquid ready-to-use formulation).

### 3.1 Bharat Biotech, India

The indigenous Indian rotavirus vaccine, ROTAVAC™ (Bharat Biotech International Ltd., Hyderabad, India), is derived from a naturally occurring human reassortant strain (G9P[11]) isolated from an Indian child [37]. The 116E strain was isolated from a maternity unit at the All India Institute for Medical Sciences (AIIMS) in Delhi and was shown to protect the neonates against subsequent rotavirus disease [38]. An international collaboration led to full development of this strain as a vaccine candidate at Bharat Biotech with initial support from US CDC and NIH [39], followed by support from PATH for the clinical development of the vaccine strain, with funding from the Department of Biotechnology (DBT), Delhi and the Bill & Melinda Gates Foundation, Seattle [40].

A Phase III randomized, double-blind, placebo-controlled, multi-center clinical efficacy study was conducted in three sites in India in 6800 infants (randomized 2:1 for vaccine to placebo) and demonstrated that the vaccine is safe and significantly efficacious in infants, with a vaccine efficacy of 53.6% against severe rotavirus gastroenteritis in the first year of life, and 48.9% in the second year [41], [42]. These results reflect similar efficacy of the commercial vaccines in developing country populations. The vaccine was licensed by the Drugs Controller General of India (DCGI) in 2014 and rotavirus vaccines were recommended for inclusion in India by the National Technical Advisory Group for Immunization (NTAGI), due to the heavy burden of rotavirus disease in India [5], [43].

The Indian government selected ROTAVAC™ for inclusion in the Universal Immunization Program (UIP) after a tender procurement process in 2015, and decided to introduce initially in four early adopter States to elucidate the early programmatic feasibility of including the new vaccine. The first generation product is a frozen formulation requiring storage at −20 °C at the central district level, although it is stable at 2–8 °C for 6 months in the downstream UIP system. The vaccine is also delivered in a 5-dose multi-dose vial with separate administrator, which was to be evaluated in the UIP in these early adopter States. After extensive training and preparation at the State and District level, the vaccine was introduced in March 2016 in the UIP systems in Andhra Pradesh, Himalchal Pradesh, Haryana and Orissa; ROTAVAC™ has been administered to over 1 million infants to date and no programmatic or safety issues have been identified [44].

Based on this early successful implementation, the Indian government has decided on the second stage of national roll-out and will include introduction into another 5 States in early 2017 (i.e. Assam, Chattisgarh, Madhya Pradesh, Rajasthan, Tamil Nadu) funded by the government and with a further introduction into Uttar Pradesh in late 2017, with Gavi-funding. Thus, by late 2017, approximately 50% of the Indian birth cohort will have access to rotavirus vaccine as part of the Universal Immunization Program [44].

The development of ROTAVAC™ continues: A subsequent clinical study evaluating the administration of ROTAVAC™ without administration of the citrate buffer used in the efficacy study was conducted and showed no diminished seroconversion in the cohort receiving no buffer [CTRI/2014/04/004548; manuscript submitted]. The “no buffer” formulation of the vaccine was licensed by the Indian regulatory agency (DCGI) during 2015, and is available in the Indian private sector since 2015. The “no buffer” formulation is also the product being implemented in the India UIP. Further clinical development is ongoing with an all-in-one liquid formulation of the vaccine (Indian Clinical Trials Registry number - CTRI/2015/02/005577).

### 3.2 Lanzhou Institute of Biological Products, China

Lanzhou Institute of Biological Products (LIBP) has developed an attenuated lamb rotavirus vaccine, which is licensed in China and is used in the private market. The monovalent lamb rotavirus strain G10P[12] was isolated in 1984 in primary calf kidney cells, from which the Lanzhou Lamb Rotavirus (LLR-85) vaccine was developed by LIBP, China [45].

The candidate vaccine received approval for Phase I and II clinical studies by the Chinese regulatory authority and were conducted in China two decades ago. In total, 640 children between 6 and 24 months of age were included in the studies (340 in the vaccine group and 300 in the placebo group). The findings from these Phase I/II studies conducted during 1992–96 indicated that the vaccine was well tolerated with fever rates comparable between vaccinees (5.9% mild and 0.6% moderate) and placebo recipients (6.7% mild and 1.0% moderate). No differences were noted between groups for diarrhea or vomiting.

A 4-fold increases in serum neutralizing antibody responses were observed in 61% of 103 vaccinees compared to 3% of 105 placebo recipients, to the LLR-85 strain and ranged between 49% and 55% for the different human rotavirus serotypes (G4-G1). However, further analysis of the subjects showed that the geometric mean titers indicated a “booster” response to the vaccination rather than a primary infection [46]. Later data on the age of acquisition of natural rotavirus infection showed that >80% of children in China are infected before 12 months of age, suggesting that many of the subjects in this trial were already antibody positive [47].

In 1998, a Phase III efficacy trial was conducted with support from international partners, including WHO which conducted three site visits to examine the production facilities and to help design the clinical trial; and the International Vaccine Initiative (IVI). Over 3000 subjects were enrolled in the randomized, blinded, placebo-controlled study, which reported 78% efficacy against rotavirus diarrhea, although only 10 cases of rotavirus diarrhea were identified in infants in the study during the first year, highlighting the lack of adequate surveillance to capture cases of rotavirus diarrhea [48]. National surveillance data in China has demonstrated over 50% of infants hospitalized with diarrhea have rotavirus in their stools [47].

Nevertheless, the vaccine is widely used in China and over 5 million doses had been distributed in the private market in China by 2012 [49]. The vaccine is currently recommended in China as a single dose annually from 2 months to 3 years of age and a booster dose between 3 and 5 years [50]. Several studies assessing vaccine effectiveness in China have reported a one-dose vaccine effectiveness of 44% (28–75%) against laboratory confirmed rotavirus gastroenteritis in children 9–11 months of age, and 77% against rotavirus related hospitalizations [49], [50]. The effectiveness was further explored in a population-based, case control study, where a protection rate of 35% (95% CI: 13–52%) was identified. A higher protection against moderate/severe rotavirus gastroenteritis caused by genotype G3 rotavirus strains of 53% (15–75%) was reported [51].

Lanzhou Institute are further developing a trivalent lamb reassortant rotavirus vaccine candidate which has recently completed a Phase III safety and efficacy study, although the results are not known at this time.

### 3.3 Center for research and production of vaccines (PolyVac), Vietnam

The government of Vietnam has encouraged a policy of self-reliance for vaccines for the local population. The Center for Research and Production of Vaccines and Biologicals (POLYVAC), Vietnam has licensed an oral, live attenuated human rotavirus vaccine, Rotavin–M1, in Vietnam [52]. Initially three candidate strains (G1P[8], G1P[4] and G4P[6]) isolated from Vietnamese infants with rotavirus diarrhea were adapted to cell culture and passaged to ascertain which strain grew to highest titer. The strains were all characterized and cell culture adaptation and preparation as a vaccine candidate followed established WHO guidelines [52]. The KH0118-2003 strain (G1P[8]) was selected based on these analyses as the best candidate for further development.

After safety was demonstrated in adults, the vaccine candidate was evaluated in a Phase II clinical study in infants to assess two different dose titers (10^6.0^ and 10^6.3^ FFU/dose) and two different administration schedules of 2 doses, 2 months apart or 3 doses, 1 month apart [53]. The comparison group received two doses of Rotarix™. There were no differences between Rotavin-M1™ and Rotarix™ in terms of solicited mild adverse events following dosing (i.e. diarrhea, vomiting, irritability or fever). The serum IgA responses to Rotavin-M1, assessed as a 4-fold increase, ranged from 51% to 63% as compared to the 58% sero-conversion of the Rotarix™ vaccine [53]. Interestingly, the highest IgA sero-conversion (73%) was for the 2-dose schedule at 10^6.3^ FFU/dose with a longer interval between doses. This increased immune response was also observed in a study with Rotarix™ given as two doses at 1-month or 2-months apart [54]. The longer interval between doses increased the IgA sero-conversion from 63% to 81% in this small study.

A Phase IIb trial using the 2-dose schedule to evaluate the safety, immunogenicity and efficacy was performed in 800 Vietnamese infants. This study showed a high IgA seroconversion (91.9%) one month after dose 2, however, the subsequent vaccine efficacy has not yet been released [NCT01502969]. The robust immune responses were sufficient for licensure by the Vietnamese national regulatory authority in 2014.

## 4 Development of new live attenuated rotavirus vaccine candidates

At present, “new generation” rotavirus vaccine candidates utilizing different strains, formulations and routes of administration are under clinical development with the aim to improve effectiveness and impact in developing countries.

### 4.1 Bovine-human reassortant rotavirus vaccine

The US National Institutes of Health (NIH) UK-Compton bovine rotavirus vaccine (UK-BRV) is a multivalent bovine-human rotavirus reassortant vaccine comprised of the G6P[5] bovine rotavirus backbone with the VP7 genes from the common human rotavirus strains incorporated as reassortants into the vaccine strains [55]. The human VP7 genes for G1 (strain D), G2 (DS-1), G3 (P) and G4 (ST-3) were established first and this quadrivalent vaccine candidate was evaluated in clinical trials showing robust immune responses to the rotavirus antigens, good safety data and non-interference with the routine childhood vaccines when co-administered [56], [57]. Importantly, the quadrivalent UK-BRV was also shown to have similar immune responses and clinical efficacy to the licensed RotaShield® vaccine in Finnish infants [58]. These results prompted the global development of the vaccine candidate; and the NIH developed further reassortants to include components for G8 (strain 1290) and G9 (strain AU32) [59] to ensure global relevance as new data emerged on circulating strains in Africa and Asia.

Non-exclusive licenses for the development and production of the UK-BRV vaccine were granted to several commercial vaccine manufacturers, including the Serum Institute of India (India), Shanta Biotechnics (India), Chengdu Institute of Biological Products (China), Wuhan Institute of Biological Products (China) and Instituto Butantan (Brazil). Funding from the Bill & Melinda Gates Foundation to PATH has coordinated vaccine development at several of these manufacturers.

Serum Institute of India (SII) has the most advanced UK-BRV program. Their first generation product is a lyophilized pentavalent vaccine containing five reassortant rotavirus strains including the G1, G2, G3, G4 and G9 components [60]. Phase I, IIa and IIb clinical evaluation was conducted in India, showing the vaccine is safe and well tolerated in adults, children and infants [60]. Serum IgA immunogenicity in infants with a dose of 10^5.6^ FFU/serotype/dose was evaluated in the Phase IIb study, giving IgA seroconversion rates of 56.7% and 60% post-dose 2 and post-dose 3, respectively [60]. Based on these results, the pentavalent vaccine was evaluated in a Phase III vaccine efficacy study in 6 sites across India, in 7500 infants randomized 1:1 to receive vaccine or placebo, and evaluated for safety, immunogenicity and efficacy (NTC02133690). This study commenced in 2015 and outcomes are predicted to be available by early 2017, with the aim to achieve licensure in India. In addition, a lot-to-lot consistency study which will also assess the co-administration of routine childhood vaccines with the pentavalent rotavirus vaccine is ongoing with results expected in 2017 (NTC02584816).

A second efficacy study was conducted by EpiCentre, Paris and Medicine Sans Frontier (MSF) in Niger including a similar number of infants and utilizing the pentavalent vaccine (NTC02145000). The results are promising and have been submitted for publication (Rebecca Grais, Director, EpiCentre, personal communication).

Shantha Biotechnics (India), Hyderabad is developing a tetravalent UK-BRV vaccine, expressing VP7 genotypes G1, G2, G2, and G4. The vaccine has been evaluated for safety and immunogenicity in Phase I and II studies [61]. The IgA seroconversion rates for the 2 highest vaccine titers (10^5.8^ and 10^6.4^ FFU/ml) were determined after administration of the third dose, with a range of 52.9–83.3%. In late 2014, a Phase III clinical trial involving 1200 6–8 weeks’ Indian infants was commenced to show non-inferiority against a currently licensed vaccine based on immunogenicity (CTRI/2014/08/004893). The results were disappointing and failed to achieve the endpoint of the study [62]. The company is not currently pursuing the vaccine development further based on competing priorities and the robust pipeline of oral rotavirus vaccines.

Butantan Institute (Brazil) has conducted a Phase I, double-blind, placebo-controlled trial to evaluate the safety and immunogenicity profile of its pentavalent UK-BRV vaccine (containing VP7 antigens G1, G2, G3, G4 and G9. In this study, 80 adult males were studied and the vaccine was found to be safe and immunogenic in this small study [63]. Difficulties to conduct further clinical trials in Brazil, where Rotarix™, GSK Biologicals is widely used in the national EPI system and has demonstrated impact on diarrhea hospitalizations [64] has hampered the continuance of the vaccine development.

Wuhan Institute of Biological Products (WIBP) (China) has commenced the development of a hexavalent UK-BRV vaccine, utilizing all six reassortants from NIH. They are still in early stage of development, having recently commenced recruitment for a Phase 1 safety trial in 2016.

### 4.2 Human neonatal rotavirus vaccine (RV3-BB)

An asymptomatic neonatal rotavirus strain isolated in Australia (G3P[6], RV3) was shown to give clinical protection in those neonates against subsequent rotavirus disease [65]. The viral strain has several unique biological characteristics including (i) clinical protection up to 3 years of age in neonates infected asymptomatically; (ii) replication in the neonatal gut even in the presence of high levels of maternal antibody; and (iii) unique heterotypic cross protection against other human rotavirus strains. The vaccine candidate is also the only strain to date that exhibits a human rotavirus VP4 P2[6] outer capsid protein which has been demonstrated to circulate widely in Africa and some parts of Asia, and is far less common in the developed world [66].

Furthermore, recent *in vitro* studies have shown binding of the VP4 surface protein to the histo-blood group antigens (HBGAs) in a genotype-dependent manner, suggesting that they are putative receptors for rotavirus binding. This may be a plausible explanation why P[6] rotavirus strains are more common in Africa, where there is a higher percentage of Lewis-negative individuals [67]. As the VP4 genotype P[8] is a component of the globally available rotavirus vaccines, the finding that Lewis-negative children are resistant to P[8] strains provides an additional potential explanation for the reduced vaccine efficacy in populations with a high percentage of Lewis-negative individuals, such as in Africa.

Birth is an established immunization time, and administration of a rotavirus vaccine could help provide the greatest opportunity to improve vaccine uptake and maximize immunization rates. Birth administration also could enhance the safety profile, as it is a time where intussusception is extremely rare. Thus, a neonatal schedule could help address some of the ongoing challenges for rotavirus vaccination programs.

Early clinical development yielded disappointing immunogenicity results [68], but the Australian research group at the Murdoch Childrens Research Institute (MCRI) have continued development increasing the titer of the vaccine candidate and adapting it to WHO-approved Vero cells. MCRI have technology transferred the vaccine strain to PT Biofarma (Bandung, Indonesia), who are developing the vaccine and optimizing the manufacturing process currently, as a vaccine for Indonesia.

The newly formulated strain (RV3-BB) is currently undergoing clinical trials in Australia, New Zealand and Indonesia. A Phase I clinical trial in Australia, revealed that the vaccine is well tolerated, safe and immunogenic in adults, children and infants [69]. A Phase IIa trial to evaluate an infant schedule (i.e. 6, 10 and 14 weeks of age) and a newborn schedule (birth, 6 and 10 weeks of age) has shown that the vaccine is robustly immunogenic using both administration schedules [70]. A vaccine ‘take’ of 93% and 90% was observed in the infant and newborn dosing schedules respectively. A Phase IIb clinical study is underway to evaluate immunogenicity and efficacy in approximately 1650 Indonesian infants is underway and results are anticipated in early 2017 (ACTRN12612001282875). Finally, a neonatal dose-ranging study is planned in Malawi to ascertain data in an African population where the VP4 P2[6] genotypes circulate commonly.

Other vaccine manufacturers in the Republic of Korea and People’s Republic of China as well as India are developing additional live attenuated rotavirus vaccines although most of these are either preclinical or very early in clinical development.

## 5 Non-replicating, parenterally delivered rotavirus vaccines

Live attenuated, oral rotavirus vaccines have proven to be generally safe and effective to prevent severe rotavirus diarrhea in infants living in developed settings. However, concerns continue about rare but severe adverse events, such as intussusception, as well as the lower vaccine effectiveness in less developed settings. WHO evaluated the risk-benefit of oral rotavirus vaccines with respect to public health impact and the rare occurrence of serious adverse events, such as intussusception and found that the risk-benefit analysis weighed heavily in the favor of rotavirus immunization [71]. Nevertheless, the occurrence of even a small risk remains a public health concern [72], and has stimulated interest in an alternative, parenteral approach to immunization based on the successes of the inactivated poliovirus vaccine.

These alternative strategies, including inactivated rotavirus particles and non-replicating rotavirus proteins, have been posed as an alternative to the current live oral vaccines [73], [74], [75], [76], [77], [78]. The proposed advantages of this approach are (i) the improved safety profile with respect to intussusception, which is believed to be triggered by the replication of the oral vaccines; (ii) a potentially improved efficacy due to circumventing the proposed interference by environmental enteropathy and maternal antibody [79]; and (iii) potentially lower manufacturing costs of the subunit rotavirus vaccine candidates.

Several non-replicating parenteral formulations are being evaluated in a range of animal models. The inactivated rotavirus particles, protein sub-units or “virus-like particles” (VLPs, structurally-similar to live virus) are being investigated as rotavirus vaccine candidates (Table 3).

**Table 3:** Non replicating rotavirus vaccines.

Vaccine	Characteristics	Developer	Development progress
Inactivated rotavirus vaccine (IRV)	Heat inactivated human rotavirus (G1P[8])	CDC, USA	Preclinical: mice/monkeys
	– Grows to high titer		Immunogenic
			Protective in animal model
			– Clinical lot prepared
			– Process development
Expressed proteins (based on VP6 inner capsid)	Truncated VP6	Cincinnati Children's Hospital Med Cent, USA	Animal studies
	VP6 combined with norovirus VLP	University of Tampere School of Medicine, Finland	– Immunogenic
			– Protected in challenge
			– Low yields
Virus like particles	Virus-like particles	Baylor College of Medicine, USA	Animal studies
	VP2/6/7; VP2/4/6/7		– Immunogenic
			– Protective small animals (predom. Homotypic)
			– Low yields/process difficult
VP8 expressed proteins (NRRV)	Trivalent truncated VP8: P[4], P[6] and P[8]	PATH, National Institutes of Health, USA	Human Phase I/II studies ongoing:
			Phase 2a immunogenicity – P[8] monovalent vaccine
			Phase 2a/b safety & immunogenicity of trivalent vaccine

### 5.1 P2-VP8^∗^ candidate, PATH

The most advanced of the non-replicating candidates is a recombinant subunit parenteral rotavirus vaccine, developed as a truncated recombinant VP8^∗^ protein of human rotavirus genotypes P[8], P[4] or P[6] expressed in *Escherichia coli* at the US NIH [80], [81]. The vaccine constructs have been demonstrated to elicit serum neutralizing immune responses in animals, and the immunogenicity could be significantly enhanced when the constructs were fused with the P2 epitope of tetanus toxoid, which elicits a strong T-cell helper function [81]. PATH, Seattle has developed the vaccine construct further, including process optimization and adsorption of the P2-VP8^∗^ with aluminum hydroxide; clinical trial lots were produced at Walter Reed Army Institute for Research (WRAIR) for the human studies.

An initial Phase I clinical evaluation of a monovalent P2-VP8-P[8] vaccine construct in healthy US adults demonstrated the safety and immunogenicity of the construct [82]. This led to an age-descending, dose-escalating study in South Africa, where the same monovalent vaccine construct was demonstrated to be well tolerated and immunogenic in children and infants in all subject age groups [83; manuscript submitted]. High levels of anti-P2-VP8^∗^ serum IgG and IgA antibody, and neutralizing antibody responses were identified in an expanded infant cohort. Interestingly, Rotarix® shedding was significantly diminished in immunized subjects versus the placebo group, suggesting that the vaccine may provide efficacy against rotavirus disease in the small intestine [83]. A Phase I/II randomized, double-blind, placebo-controlled, dose-escalation, descending-age trial is currently underway to determine safety and immunogenicity of the trivalent P2-VP8-P[8]/P[6]/P[4] vaccine in South African adults, children, and infants (NCT02646891); results are expected in late 2017.

### 5.2 Inactivated rotavirus vaccine, CDC

An inactivated whole virus approach is being developed by US Centers for Disease Control and Prevention (CDC, USA). The CDC-9 vaccine replicates well in Vero cells, is fully inactivated using a thermal approach, including maintenance of the triple-layered virions after downstream purification steps [84]. When the inactivated CDC-9 constructs were adjuvanted with aluminum hydroxide and administered intramuscularly in mice, a robust immune response and protection from oral challenge was observed [85]. In addition, studies in mice with the monovalent CDC-9 strain (G1P[8]) demonstrated some degree of heterotypic immunity against other human strains in gnotobiotic piglets and guinea pigs, although low to no immunity to a bovine-human reassortant strain or the parent bovine strain (WC3); the study concludes that 3 doses of the vaccine construct give enhanced immune responses showing a dose-dependent response, and increased the IgG avidity index of these responses [86].

Various delivery approaches have been proposed, including intramuscular described above, and an intradermal using a microneedle approach, which did indicate a robust immune response even with a fractionated dose [87]. The developers of the vaccine are planning first-in-human studies and actively seeking a manufacturing partner for future development and marketing of the vaccine construct.

### 5.3 VLPs and other subunit approaches

Various other approaches to the design of subunit rotavirus vaccines have been proposed including viral-like particles (VLPs) [88] in various formats – usually the inner capsid VP6 antigen, with or without the outer capsid proteins VP4 and/or VP7, and in some platforms combined with norovirus VLPs [89]. In preclinical studies, most of these constructs do elicit strong immune responses in mice and offer protection against oral challenge with rotavirus strains.

The VP6 protein expressed as a chimera with maltose binding protein (MBP:VP6) has also demonstrated both immunogenicity and protection when administered via various routes including intra-nasally, intra-rectally or orally to mice [90]. When challenged with rotavirus, vaccinated mice were nearly 100% protected from fecal shedding of rotavirus, which was dependent on co-administration of an effective adjuvant. Protection was stimulated by only 1 dose of MBP:VP6, remained fully intact for at least 1 year, was effective in all strains of mice tested, and could also be effectively delivered orally or intra-rectally. In contrast to live rotavirus vaccines, CD4(+) T cells were found to be the only lymphocytes required for protection. If VP6 elicits comparable protection in humans, it would represent a potential second-generation vaccine candidate.

Both these approaches are in early pre-clinical R&D and have not yet addressed issues such as manufacturing process or scale up and first-in-human studies.

## 6 Summary

Thus, 10 years after rotavirus vaccine introduction has occurred in many countries, their public health impact has been demonstrated by reductions in rotavirus-associated mortality and diarrheal hospitalizations in all socio-economic settings. In low income settings, the lower vaccine effectiveness and the early indications that rotavirus vaccine protection is not as enduring beyond the first year of life, pose ongoing challenges to sustainability of early vaccine successes. New live oral and non-replicating rotavirus vaccine candidates continue to be developed with the aim to improve effectiveness in developing country settings, by enhancing vaccine coverage with new vaccines expanding the global supply, and by improving efficacy and safety of the vaccines. Only time will allow us to answer whether these new generation rotavirus vaccines will improve vaccine effectiveness and impact for those who need vaccines the most.
